# PReS-FINAL-1019: Inflammatory monocytes induce resistance of effector t cells to suppression

**DOI:** 10.1186/1546-0096-11-S2-P16

**Published:** 2013-12-05

**Authors:** EJ Wehrens, A Boltjes, M Klein, SJ Vastert, BJ Prakken, F Van Wijk

**Affiliations:** 1 Department of Pediatric Immunology, University Medical Center Utrecht, Utrecht, The Netherlands

## Introduction

Ever since their discovery research has focused on whether deficiencies in FOXP3^+ ^regulatory T cells (Treg) underlie human autoimmune pathology. Very recently however, the topic of Treg extrinsic factors as the cause of regulatory defects in chronic autoimmune inflammation has become more prominent in the discussion. It has become clear that resistance of effector cells (Teff) to suppression contributes to disturbed immune regulation in autoimmune inflammation, especially at the site of inflammation. Therefore, targeting this unresponsiveness to suppression could be a promising treatment option for patients with autoimmune disease. It remains unknown how resistance of T cells to suppression is induced.

## Objectives

To investigate how resistance of Teff cells to suppression is induced, and what the role is of antigen-presenting cells (APC) in this.

## Methods

We phenotypically characterized APC present at the site of autoimmune inflammation in patients with juvenile idiopathic arthritis (JIA) by means of flow cytometry and Luminex technology. Furthermore, we co-incubated APC with Teff and Tref, and subsequently measured Teff proliferation and cytokine production to investigate the role of APC in inducing Teff resistance to suppression.

## Results

We observed a clear difference in the composition of APC in synovial fluid (SF), obtained from inflamed joints, compared to peripheral blood. Moreover, SF monocytes displayed strong pro-inflammatory characteristics with especially high TNFa and IL-6 production directly *ex vivo*. Upon co-culture with Teff, these SF monocytes and not dendritic cells (DCs) induced unresponsiveness of Teff to suppression, resulting in impaired Treg-mediated control of cell proliferation and cytokine production. By blocking IL-6, TNFα or both, control of Teff proliferation and cytokine production by Treg was (partially) restored in these co-cultures.

## Conclusion

These data shed new light on the role of monocytes in autoimmune pathology, indicating that monocytes actively contribute to the ongoing inflammation by interfering with T cell regulation. Moreover, our results identify inflammatory monocytes and their ability to induce resistance to suppression as a new target to treat autoimmune inflammation.

## Disclosure of interest

None declared.

